# Balneotherapy and Manual Therapy of Key Myofascial Trigger Points as Therapeutic Integration for COPD Associated with Myofascial Pain Syndrome: A Case Series

**DOI:** 10.3390/healthcare14060788

**Published:** 2026-03-20

**Authors:** Giovanni Barassi, Maurizio Panunzio, Loris Prosperi, Celeste Marinucci, Antonio Moccia, Davide Pio Fratta, Floriana Cristinziano, Michele Pio Della Rovere, Pier Enrico Gallenga

**Affiliations:** 1 Center for Physiotherapy, Rehabilitation and Re-Education (Ce.Fi.R.R.), Venue “G. d’Annunzio” University of Chieti-Pescara, 66100 Chieti, Italy; panunzio.maurizio76@gmail.com (M.P.); loris.prosperi@riabilitazioneunich.it (L.P.); celeste.marinucci@riabilitazioneunich.it (C.M.); miki.dellarovere@gmail.com (M.P.D.R.); pier.enrico.gallenga@riabilitazioneunich.it (P.E.G.); 2 Castelnuovo della Daunia Thermal Medicine Center, 71034 Castelnuovo della Daunia, Italy; antoniomocciaam5@gmail.com (A.M.); davide.fratta1999@gmail.com (D.P.F.); floriana_82@hotmail.com (F.C.)

**Keywords:** chronic obstructive pulmonary disease, balneology, manual therapy, trigger points, physical therapy, rehabilitation

## Abstract

**Background**: Chronic Obstructive Pulmonary Disease (COPD) is a common condition that can cause dyspnea, pain, and biomechanical-postural alterations, especially when overlapping with Myofascial Pain Syndrome (MPS). Balneological rehabilitation medicine can help manage COPD and MPS, but it lacks homogeneity and detailed descriptions of effective therapeutic protocols. Therefore, we conducted a case series to preliminarily evaluate the clinical effects of a detailed and codified approach, called Bio-Physico-Metric Integrated Thermal Care (BPM-ITC), for COPD+MPS. **Methods**: 10 patients were observed while undergoing 20 sessions of BPM-ITC in 4 weeks. Patients were assessed before and after the protocol using the Medical Research Council (MRC) dyspnea scale, Numeric Pain Rating Scale (NPRS), and the Bio-Postural Questionnaire (BPQ) for bio-physical health status. Treatments included manual therapy of key myofascial trigger points combined with crenotherapy, steam inhalations, mud therapy, vascular path, and water-based motor re-education. **Results**: At the end of the protocol, clinically relevant improvements were observed in almost all parameters considered in single observed cases; overall statistical analysis of the data highlighted significant positive effects in concomitance with the BPM-ITC protocol. **Conclusions**: The BPM-ITC protocol was followed by significant clinical improvements in the observed cases, suggesting its potential as a complementary approach for COPD+MPS. Further studies on this topic are recommended.

## 1. Introduction

Chronic Obstructive Pulmonary Disease (COPD) is a rather widespread pathology, hitting about 300 million people worldwide [1], and it is estimated that it could become the third most prevalent cause of death over the next 30 years [2]. It is also estimated that up to 80% of people actually affected by the disease have it in an insidious and/or not yet diagnosed form [3]. COPD is a condition characterized by obstructed ventilatory patterns, often slowly progressing and only partially reversible, mostly due to environmental factors, such as smoking and genetic predisposition, whose levels of influence, however, have not yet been fully defined and clarified [1]. The diagnosis is mostly clinical, based on the presence of dyspnea, chronic cough or mucus production, and a personal history of exposure to risk factors, but it usually also requires a postbronchodilator FEV1/FVC value of less than 0.7 at the forced spirometry test [4].

Treatment for COPD is complex, depending on the severity of the condition, and it is primarily based on inhaled bronchodilator drugs, followed by oxygen therapy and, in extreme cases, lung surgery [5]. Furthermore, both the removal of any modifiable risk factors (e.g., smoking cessation therapy) and the application of pulmonary rehabilitation protocols aimed at increasing the patient’s breathing capacity are often recommended [5].

Among the complementary and non-invasive approaches to COPD, we also find balneotherapy, which uses thermal waters and their derivatives to exploit a series of therapeutic techniques that appear to be beneficial for the pathology [6]. At the same time, balneotherapy appears to be useful in improving the health of patients suffering from various musculoskeletal problems, such as fibromyalgia and Myofascial Pain Syndrome (MPS), typically related to the presence of Myofascial Trigger Points (MTrPs) [7,8]. Manual therapy techniques also appear to be valid adjuvant therapies in the management of both COPD [9] and MPS associated with MTrPs [10].

A commonly underestimated aspect of COPD management is the ability of the condition to negatively affect the musculoskeletal system, often associated with the presence of myofascial pain, especially in areas of the body such as the cervical and lumbar spine, chest, and diaphragm [11,12], all of which are involved in the respiratory functions of the body and could benefit from the application balneotherapy and manual therapy approaches. However, the current research in the field of balneotherapy and manual therapy is lacking in methodological quality and homogeneity, just as there is a lack of studies regarding the combined application of manual and balneological therapies in the context of coexisting COPD and MPS. This leaves room for exploration of approaches combining both balneotherapy techniques and manual therapy of MTrPs in a structured and detailed way, and according to a personalized and repeatable pattern.

An approach that, in our experience, has proven effective in combining the therapeutic properties of balneotherapy and manual therapy is the one defined as Bio-Physico-Metric Integrated Thermal Care (BPM-ITC). In previous observational studies, we have found that the approach is associated with improved health in various contexts, such as oncology [13] and neuromuscular conditions [14]. Consequently, it could be hypothesized that the BPM-ITC approach could potentially fill the research gap regarding the role of balneotherapy and manual therapy in the management of COPD associated with MPS.

Therefore, in order to assess if the therapeutic potential of the BPM-ITC approach can be extended to patients affected by COPD+MPS, we conducted a study to preliminarily observe the clinical progression and feasibility of the protocol regarding dyspnea, pain, and the bio-postural health status in patients united by this specific comorbid condition.

## 2. Materials and Methods

### 2.1. Study Design and Ethics

This research is a case series carried out from May to June 2025 at the “Castelnuovo della Daunia Thermal Medicine Center” (Castelnuovo della Daunia, Italy), in cooperation with the staff from the “Center for Physiotherapy, Rehabilitation and Re-Education—Ce.Fi.R.R.” (Chieti, Italy). All participants received safe, non-invasive treatments in accordance with national safety protocols. The study was open to any patient who did not present contraindications during their initial medical screening. Conducted in line with the Declaration of Helsinki and Good Clinical Practice standards [15], the study required written informed consent from all participants, which was obtained in advance from each patient. This practice is in accordance with the operational routine of the study venue, aimed at the possibility of retrospectively analyzing clinical data from any willing patient who attended the site, for observational research purposes. Data were extracted from medical records filled and maintained by specialists in physical medicine and rehabilitation, or rheumatology. While at the national level there is a lack of regulatory clarity regarding the competences and requirements for approval by an ethics committee of retrospective non-pharmacological studies [16], this research adhered to strict ethical standards under the ISO 9001:2015 [17] certification (no. IT15/0304) issued to the Ce.Fi.R.R. institution by the national accreditation body ACCREDIA. The study complies with the PROCESS Guidelines adaptation for case series about non-surgical intervention [18].

### 2.2. Inclusion and Exclusion Criteria

The cases presented were collected from all patients attending the study venue during the observation period. Specifically, all patients diagnosed with moderate/severe COPD+MPS, confirmed by rheumatologists and physical and rehabilitation medicine physicians of the study venue based on a clinical history of COPD previously diagnosed outside the facility associated with the presence of localized and radiating musculoskeletal pain in the presence of MTrPs, were consecutively considered. During the same period, as per the standard practice of the study center, all patients with cardiac or renal failure, severe hypertension, severe thrombophlebitis, acute phases of rheumatic diseases, immunodeficiency, open wounds and ulcers, psychiatric disorders, severely limited walking ability, and active infections were excluded from treatment. Therefore, clinical data were collected from medical records of 10 COPD+MPS patients (4 males and 6 females, mean age 69 ± 7 years) who attended the Castelnuovo della Daunia Thermal Medicine Center.

### 2.3. Evaluation Methods

The clinical status of patients was monitored through the following assessment tools, applied before (T0) and after (T1) the execution of the entire treatment protocol:

- Medical Research Council (MRC) dyspnea scale: a 5-point scale that assesses the level of dyspnea subjectively perceived by the patient in relation to different types of physical exertion [19]. The lowest level (grade 0) indicates dyspnea only with intense physical exertion, while the highest level (grade 4) indicates dyspnea even at rest. The scale is useful for assessing the level of respiratory disability in patients with COPD, identifying dysfunctions requiring particular attention with a cutoff grade of ≥2 [19].
- Numeric Pain Rating Scale (NPRS): a common tool for assessing the intensity of pain subjectively experienced by patients, derived from the ten-level Visual-Analog Scale (VAS) [20,21]. Observed patients were required to express a value from 0 to 10 corresponding to the maximum level of pain perceived at the musculoskeletal level, especially thinking of the body area representing the most insidious point for them. The NPRS score specifically defines no pain if equal to 0, mild pain if between 1 and 3, moderate pain if between 4 and 6, severe pain if between 7 and 9, and worst pain ever endured if equal to 10. The tool is also useful for assessing the levels of pain in COPD and MPS [20,21].
- Bio-Postural Questionnaire (BPQ): a set of questions investigating the general health status of an individual quickly and comprehensively. It investigates the state of health at the musculoskeletal level (such as specific pain, swelling of the limbs, perceived instability), visceral level (such as digestion, renal activity, cardiovascular system, genito-hormonal system), perceptual level (such as vision, hearing, central and peripheral sensitivity), psycho-emotional level (such as anxiety, attention, stress) and lifestyle level (such as smoking, physical activity, pharmacological and rehabilitative therapies). It is composed of 57 questions, for which it is possible to give an answer with an assigned value ranging from 0 (no dysfunction reported) to 2 (maximum dysfunction reported), except for two responses that can have a maximum dysfunction value of 1. By making the sum of the values of each response, a score of the biological and postural dysfunction of the patient can be obtained, ranging from 0 (no bio-postural dysfunction) to 112 (maximum bio-postural dysfunction). The questionnaire is self-completed by the patient and therefore allows both the collection of some anamnestic data and the quantification of the level of overall bio-postural dysfunction for evaluation and monitoring purposes.

### 2.4. Treatment Protocol

The BPM-ITC protocol utilizes the BPM assessment approach to guide the application of various balneotherapeutic techniques based on musculoskeletal findings. During each session, clinicians identify the most dysfunctional myofascial regions to locate Key Myofascial Trigger Points (KMTrPs). Following the BPM methodology, KMTrPs are identified by the presence of palpable painful nodules, restricted range of motion, and visible positional asymmetry in a district of the body. These points are recognized as primary drivers of functional, postural, and visceral imbalances, manifesting both locally and systemically. Specifically, bilateral assessments were performed on a comprehensive set of muscles, including the pectoralis major, rectus abdominis, rectus femoris, adductor magnus, tibialis anterior, adductor hallucis, quadratus plantae, and abductor digiti minimi. The evaluation also extended to the splenius cervicis, upper and lower trapezius, paraspinal longissimus dorsi, quadratus lumborum, gluteus maximus, gastrocnemius, soleus, and hamstrings. Within the ITC framework, approximately three KMTrPs were identified per patient in each session to receive targeted treatment. KMTrPs were assessed in each patient at the beginning of each individual session of the protocol by the same professional balneotherapists in charge for their treatment. These brief evaluations, lasting approximately 5 min, were carried out in an isolated environment to ensure privacy and accuracy. The diagnostic process integrated a standardized sequence of visual observation, manual palpation, and active range of motion assessments, systematically performed across three primary postural orientations: standing, supine, and prone. Assessments focused on the key muscles and anatomical areas listed above.

**Figure 1:**
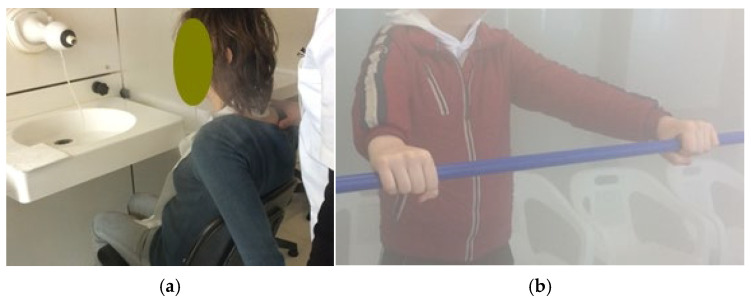
(**a**) Example of patient positioning for Thermal Water Inhalation: note the warm-humid steam jet coming out of the nozzle positioned 50 cm from the patient, with the therapist applying thumb pressure to a KMTrP located in the right levator scapulae of the patient; (**b**) Example of the upper limb flexion exercise performed by the patient in coordination with breathing cycles.

**Figure 2:**
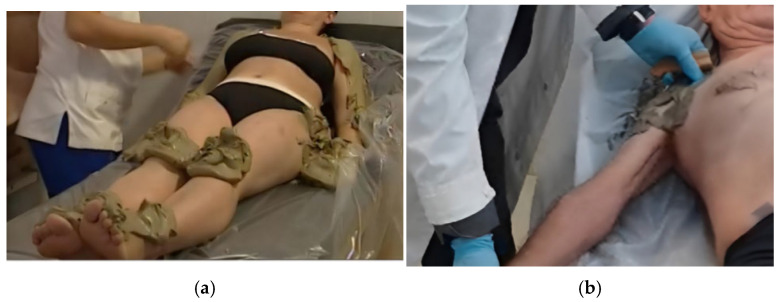
(**a**) Example of Mud Therapy application process: it is possible to see that the therapist is applying the thermal mud on all the major joints of the patient, to then proceed with the application on the KMTrPs identified during the BPM assessment; (**b**) A detail of an example of the tool-assisted manual treatment of the left pectoralis major KMTrP during the Mud Therapy phase.

**Figure 3:**
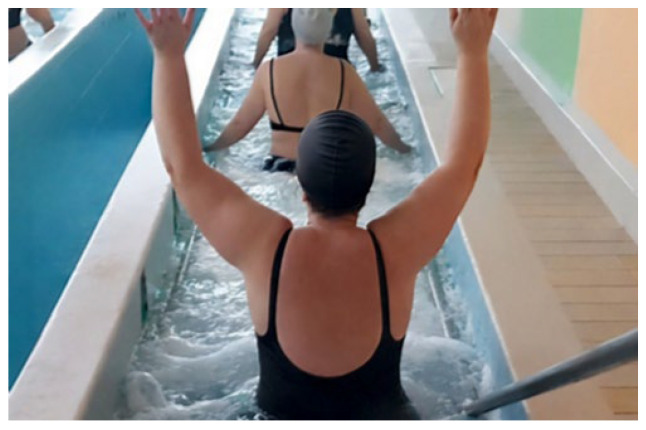
Example of patients doing the Ozonated Vascular Circuit and the upper limb flexion exercise.

**Figure 4:**
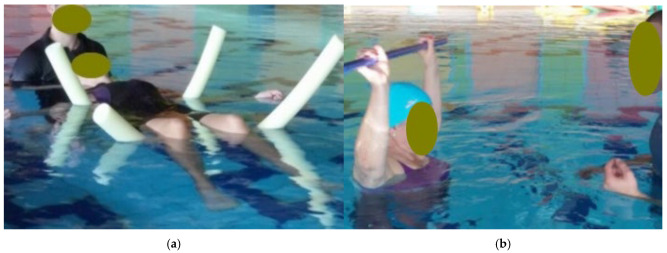
(**a**) Example of a patient floating coordinated with diaphragmatic breathing, which will be followed by pendulum movements supported by the same pool noodles positioned at the level of the spine; (**b**) Example of upper limbs flexion exercise with a stick performed in the pool.

**Figure 5:**
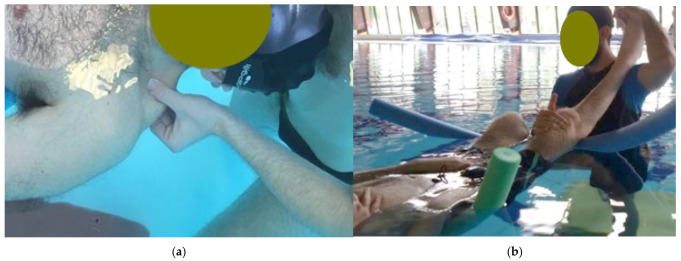
(**a**) Example of the manual treatment of a KMTrP of the left upper trapezius using a pinch technique performed in water with the patient floating; (**b**) Example of lower limb mobilization, specifically for the right ankle and knee, performed in water with the patient floating.

**Figure 6:**
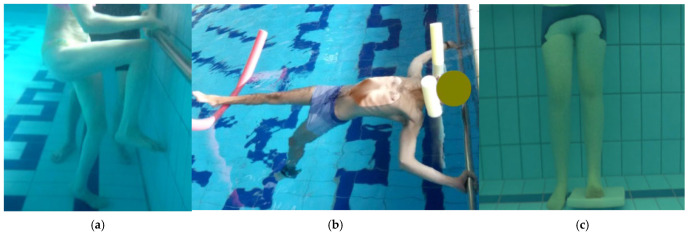
(**a**) Example of lower limb flexion-extension exercise at the barre; (**b**) Example of dynamic self-stretching of the left ileo-psoas muscle and bilateral pectoralis major with support pool noodles; (**c**) Example of plantar dorsiflexion with the tilting tablet, performed in water.

**Figure 7:**
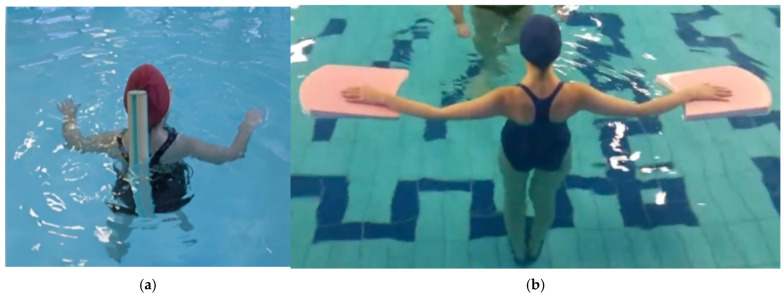
(**a**) Example of pedaling exercise performed with the support of a pool noodle; (**b**) Example of the starting position for the lateral swimming exercise performed with the support of tablets.

The ITC protocol was implemented through a sequential series of the following balneotherapeutic techniques during every session:

- Hydroponic Therapy (Crenotherapy): This procedure involved the oral administration of approximately 500 mL of bicarbonate-sulfate-alkaline-earth mineral water sourced from the “Castelnuovo della Daunia Thermal Medicine Center.” Each subject consumed the water after a minimum six-hour fast. To optimize the session timeline, patients were permitted to drink the water while concurrently receiving other ITC treatments (using a small bottle marked with the name that they carried with them during the following treatments).
- Thermal Water Inhalation: This phase involved the administration of a warm-humid steam jet via a nozzle positioned 50 cm from the patient. The thermal water was delivered as a uniform mist at 1.5 atmospheres of pressure and a temperature of 37–38 °C for a total of 20 min. During the first 10 min, a therapist positioned standing behind the seated patient performed manual stimulation of the previously identified KMTrPs and respiratory-related muscles (including the diaphragm, pectoralis minor, levator scapula, and the stellate ganglion area) [Figure 1]. These digital-pressure and massage techniques lasted between 30 s and 1 min per point, eventually accompanied by assisted static stretching maneuvers. In the final 10 min, patients performed upper limb flexion exercises using a wooden or plastic stick held at shoulder width [Figure 1]. These movements were coordinated with breathing cycles (4 s deep inhalation/expansion and 4 s forced exhalation), alternating one minute of activity with one minute of rest.

- Mud Therapy: This stage involved the application of matured bentonite thermal mud directly to the main joints of the body (shoulders, elbows, wrists, spine, hips, knees, and ankles) and the previously identified KMTrPs [Figure 2]. While the patient was supine on a medical bed, the mud was applied for 15 min, followed by a 15 min individual bath featuring thermal water and ozone hydromassage jets. Subsequently, patients underwent a 20 min rest period in a temperate environment. During the first 10 min of this rest phase, a therapist applied manual stimulation to the KMTrPs using the same massage and digital-pressure techniques employed during the inhalation therapy, eventually helping himself with a metal and wooden T-bar massage tool to avoid direct contact with the mud for hygienic reasons [Figure 2].

- Ozonated Vascular Circuit: This procedure involved walking between two thermal water pools maintained at contrasting temperatures (24 °C and 38 °C) to stimulate venous and lymphatic circulation in the lower extremities. The 15 min session required patients to alternate between the pools every minute. Both pools utilized ozonated water jets at 4–6 atmospheres of pressure to provide a proprioceptive stimulus. During the walk, patients repeated the upper limb flexion exercises introduced during the inhalation phase, in this case without the wooden/plastic stick [Figure 3].

- Water-based Re-education: This phase involved therapist-assisted exercises and mobilizations in a warm thermal pool (32.5 °C). Each 60 min session was structured into four distinct 15 min stages. The first stage, “Environmental Adaptation and Relaxation”, focused on general exercises including floating coordinated with diaphragmatic breathing and pendulum movements supported by tablets or pool noodles positioned at the level of the spine and walking in coordination with the breathing cycle, followed by the upper limbs flexion exercise with a stick described in the previous phases of the protocol [Figure 4]. The second stage, “Myofascial Stimulation”, utilized manual digital pressure on identified KMTrPs and passive mobilization of the spine and limbs while the patient floated [Figure 5]. The third stage, “Articular Mobility and Proprioception”, consisted of active lower limb flexions and extensions at the barre, dynamic self-stretching with support tablets or pool noodles, and bilateral plantar dorsiflexion with a tilting tablet [Figure 6]. The final stage, “Active Body Stabilization”, involved pedaling and lateral swimming exercises supported by pool noodles and tablets [Figure 7].

The therapeutic protocol consisted of 20 BPM-ITC sessions, administered in five-day cycles over a four-week period. The protocol was applied in its entirety, including all its components and techniques, as described, to all patients observed, with only minimal adaptations to the execution technique of water-based exercises depending on the individual motor skills of each patient. Treatments were performed by professional balneotherapists of the thermal medicine center where the study was conducted. As the data observed were obtained from the medical records of the patients, pre- and post-BPM-ITC clinical assessments were made by medical doctors of the study venue, specialized in physical medicine and rehabilitation or rheumatology, who are generally responsible for the clinical assessments and treatment prescriptions of all patients attending the thermal medicine center.

### 2.5. Data Presentation and Statistical Methods

As a case series, the primary objective of the study is to quantitatively present the data relating to the observed subjects. However, to obtain information merely on the overall trend of the clinical values detected in the study patients, although not aimed at the generalization of the results, a basic pre-post interference statistical analysis was also conducted. Statistical analysis was performed through the Statistics Kingdom online calculator (www.statskingdom.com, accessed on 27 January 2026). Due to the small size of the sample (n = 10) composed of the single cases of the series, analysis was performed through the non-parametric Wilcoxon signed-rank test for dependent samples. Significance level was set at *p* < 0.05. The effect size (r) and interquartile range (IQR) were also calculated.

## 3. Results

Baseline relevant demographic and clinical characteristics of the 10 cases observed are shown in Table 1.

All patients (Pt.) originated from the geographical area where the study took place. None of the patients reported smoking at the start of treatment. Patients reported no regular physical activity, with the exception of cases 3, 6, and 7, who reported sporadic physical activity (less than twice a week). All patients followed personalized drug therapies for all their respective comorbidities; importantly, personal pharmacological regimens were maintained without modification for the entire duration of the study, under the strict supervision of the clinical staff and in accordance with the standard operating procedures of the study venue.

A comparison of the primary evaluation metrics recorded at T0 and T1 reveals a consistent reduction in scores for every patient following BPM-ITC treatment (Table 2). Statistical analysis of the sample, ideally composed of all individual cases, demonstrated significant clinical improvements across all scales, specifically a 41.6% average decrease in MRC, a 35.1% average reduction in NPRS, and a 39.4% average drop in BPQ scores (Table 2). It should be highlighted that, given the small sample size, the statistical significance detected for all variations should be interpreted with caution.

Analyzing the clinical evolution of individual patients, it can be noted that all of them, regardless of sex, BMI, and comorbidities, showed a reduction in the dyspnea value measured with the MRC scale equal to 1 point (Table 2).

About perceived pain assessments, it is possible to note how patients 1, 2 and 10, sharing characteristics like male sex and high BMI (≥29, i.e., from markedly overweight to obese onwards), all showed the maximum NPRS value (10 points) before undergoing BPM-ITC treatment (Table 1) and, at the same time, the largest improvements in the same parameter, equal to 4, 5 and 4 points respectively, after the end of the protocol (Table 2). However, this apparent correlation trend between male gender, high BMI, and large improvement in NPRS value would not appear to occur when the initial perceived pain value is not equal to or close to the maximum possible score for the scale. In fact, patient 9, a male with a high BMI (Table 1), had an initial NPRS value of 6.5, which was reduced by only 0.5 points after the BPM-ITC treatment, corresponding to the lowest NPRS reduction in the entire sample (Table 2). Furthermore, the overall observation of the sample would seem to indicate a drop in the dispersion of the NPRS score between T0 and T1, since the reduction in the IQR of this parameter could indicate a convergence of the pain level towards moderate values (between 4 and 6) in response to the BPM-ITC treatment even for cases with initial pain values considered severe (7 or above) (Table 2).

Among all the individual cases, patient 5 presented the second lowest reduction in the NPRS value between T0 and T1, corresponding to 1.5 points (Table 2); the same patient 5 is also the one who achieved the lowest improvement in terms of BPQ Score (equal to only 2 points) between T0 and T1 (Table 2). It can be noted that patient 5 was also the one who had among his comorbidities a musculoskeletal condition clearly reported as severe (polyarthritis) (Table 1).

BPQ Score also improved in every single case observed between T0 and T1 (Table 2), although the improvements in this parameter appear more random and less linear than those of the other clinical values, apparently not very consistent even with variables such as sex, age, BMI, and comorbidities. This aspect would seem to be confirmed by the increase in the BPQ Score IQR between T0 and T1, which could reflect an increased dispersion of the score, despite the observed improvements of the parameter in every single case (Table 2).

All participants adhered to the scheduled treatment sessions. No adverse events were reported, either verbally or in writing, during the observation period, and the study protocols remained unchanged throughout the same period of time.

## 4. Discussion

This case series provides preliminary evidence that BPM-ITC intervention seems to be correlated with reductions in dyspnea severity and musculoskeletal pain, alongside improvements in the bio-postural health status of patients presenting with comorbid COPD and MPS, with some apparent peculiarities emerging in some individual cases, in the presence of variables such as high initial level of pain, severe initial impairment of the musculoskeletal system and the presence of significant overweight.

Regarded as a cornerstone of traditional rehabilitative medicine, balneotherapy remains a highly effective adjunct modality for addressing the clinical complexities of both COPD and MPS [6,7,8]. Balneotherapy, in different forms, appears to be very effective in improving lung function in patients suffering from dyspnea, including in cases of COPD [22]. At the same time, spa treatments appear to be a valid therapeutic support in improving function and reducing pain in a multitude of musculoskeletal system conditions, such as MPS [23]. However, research in the field of balneotherapy is strongly influenced by extreme methodological heterogeneity [24], which can only be bypassed through proposals for detailed, standardized, and replicable protocols [25], as occurs in the case of the BPM-ITC observed in the present case series. Despite the documented clinical efficacy of balneotherapy, the specific contributions of thermal water constituents and concurrent rehabilitation exercises remain to be fully characterized. The implementation of codified, personalized protocols like BPM-ITC might be a critical step toward establishing rigorous, evidence-based standards in contemporary balneology [26,27].

Considering the absence in our protocol of direct evaluations of the eventual physiological effects induced by BPM-ITC treatment in the observed patients (such as blood and histochemical analysis), we can just rationally speculate that the favorable outcomes observed may be attributed to the synergistic integration of mechanical and physio-metabolic stimuli elicited by thermal water, which acts as a therapeutic medium and facilitator for the manual interventions within the BPM-ITC protocol. Crucially, the identification of dysfunctional KMTrPs serves as the physiological gateway for modulating neuro-visceral convergence pathways. These convergence phenomena facilitate the transmission of musculoskeletal and visceral symptomatology, mediated primarily through the posterior horn of the spinal cord and potentially involving hypothalamic paraventricular structures [28,29].

Thermal mineral waters, administered via ingestion, inhalation, or direct tissue contact, exhibit significant anti-inflammatory properties, modulating key mediators such as pro-inflammatory cytokines and Tumor Necrosis Factor [30,31]. Specifically, sulfur-rich varieties have been identified for their antioxidant, cytoprotective, and anti-inflammatory efficacy across both systemic and transdermal delivery routes [32,33]. An expanding body of evidence highlights the pivotal role of sulfur, specifically hydrogen sulfide, as a key signaling molecule in modulating cellular homeostasis [34]. These findings underscore its significant therapeutic potential across a diverse range of physiological and pathological contexts [34]. Furthermore, bentonite-based thermal muds contribute to therapeutic outcomes through detoxifying mechanisms, bacteriostatic activity, and the exchange of biocompatible minerals at the application site [35,36]. Evidence suggests that prolonged pelotherapy reduces Prostaglandin E2 and Leukotriene B4 levels, modulates cartilage-degrading cytokines, and sequestrates TNF [37,38]. Recent scientific advancements have focused on characterizing the unique microbiome of thermal waters, muds, and peloids. A robust body of preclinical and clinical research has elucidated the significant anti-inflammatory, antioxidant, immunomodulatory, and regenerative properties of bioactive metabolites synthesized by these specialized microbial communities [34]. Within the BPM-ITC protocol, these multifaceted biochemical properties of thermal mineral waters, likely attributable also to the bicarbonate-sulfate-alkaline-earth mineral water of the thermal medicine center where the study was conducted, could have determined the improvements in the clinical condition of the observed cases; this is probably due to the bioactive properties of these specific waters, presumably similar to those described so far for waters with a sulphurous composition, combined with a synergistic enhancing effect between the exposure in multiple ways to these thermal waters and the manual techniques applied during the various phases of the treatment.

The exploitation of thermal water as a medium for motor activity and proprioceptive stimulation, specifically during ozonated vascular therapy and aquatic re-education, likely augmented the therapeutic outcomes. The intrinsic physical properties of the aquatic environment, including thermal regulation, buoyancy-induced microgravity, hydrostatic pressure, and viscosity-dependent resistance, are fundamental to the efficacy and versatility of motor recovery protocols [39]. Consequently, thermal water serves as a highly adaptable and secure substrate for the rehabilitation of motor, postural, and cardiorespiratory functions across a spectrum of musculoskeletal, respiratory, and visceral pathologies [40].

Since COPD and MPS are both characterized by chronic states of mechanical dysfunction and tissue inflammation at the pulmonary [41,42,43] and musculoskeletal [44,45] levels, the ability of a protocol such as the observed BPM-ITC to act simultaneously on the mechanical and physio-metabolic aspects of the pathologies may have been the key to the positive results observed.

In relation to the clinical evolution of patients undergoing BPM-ITC, it is essential to emphasize that, observing the cases both individually and as a hypothetical sample, the clinical improvements are evident and the trend of improvement appears consistent, although the amount of data available is not sufficient to exclude the influence of placebo or Hawthorne effects or variations caused by the natural clinical history of the patients.

Specifically, all MRC values showed a pre- to post- BPM-ITC treatment reduction ≥ 1 point, consistent with the minimally clinically important difference typically associated with MRC in the context of rehabilitation [46]. In this regard, it is important to underline that dyspnea is a symptom that naturally tends to worsen linearly over time in COPD patients with a stable clinical-therapeutic situation [47], therefore the fact that each individual case reported a significant reduction in the MRC score would seem to suggest that the protocol may mitigate the typical symptomatic decline in this cohort, at least in the short term. However, in the absence of a control group, these preliminary observations should be interpreted with caution and require validation through controlled trials. Anyway, this effect seemingly occurred in a generalized way in all cases observed, regardless of variables such as age, BMI, comorbidities, and starting MRC score.

A similar situation can be described for the pre- to post- BPM-ITC treatment NPRS scores, which, except for patients number 5 and 9, decreased by a value ≥ 2 points, also consistent with the minimally clinically important difference typically associated with the NPRS in the presence of chronic musculoskeletal conditions [48]. By comparing the individual case reports, we also noted that there may be correlations between variables such as gender, BMI, and severity of comorbidities, and levels of perceived pain and biophysical health status. This aspect deserves further exploration in future research on the topic, using larger and more homogeneous samples to allow for stratified assessments. In fact, even with regard to the aspect of pain, given the small number of cases observed in the current study and the absence of a control group, it is not possible to draw definitive conclusions on these apparent trends.

Despite the positive findings, which would seem to suggest at least a potential therapeutic role for the BPM-ITC approach in COPD+MPS patients, this study presents multiple limitations due to its short-term retrospective case-series design.

The number of cases is relatively small, as well as heterogeneous in terms of BMI, comorbidities, and severity of the initial primary condition, preventing the possible generalization of the results and drawing definitive conclusions. It should be noted that, since the study involved retrospectively observed cases, there was no prospective selection of patients to be studied, so basically all cases of COPD+MPS that converged at the study site in the indicated period were considered. It should be noted, however, that the 10 reported cases were collected by the study center over a relatively short period of time (realistically just under two months) and presented the co-occurrence of two overlapping primary conditions (COPD+MPS). Therefore, given the consequential and retrospective selection process, the number of cases collected in relation to the observation time in a single-center setting represents the maximum possible for the study site, although most likely not representative of the entire population of COPD+MPS patients and not free from forms of selection bias.

Regarding the generalizability of the results, as previously stated, it must be considered that the lack of control cases, both in the form of sham treatment and standard treatment only, as well as the absence of long-term follow-up assessments after the completion of the BPM-ITC treatment protocol, constitutes a further significant limitation of the study. These aspects are also due to the observational nature of the study, as well as the impossibility of observing untreated pathological cases outside of those who turned to the study center for rehabilitation with a balneological approach, for ethical reasons of non-denial of requested treatments.

Finally, the inherent subjectivity of exploited clinical scales imposes significant constraints on current assessment tools. In fact, due to the retrospective design of the case series, it was possible to base the observation exclusively on the rapidly fillable and non-invasive assessment scales routinely present in the medical records of all patients attending the study center, without the possibility of prospectively integrating assessments specific to the study. Furthermore, more specific and invasive assessment tools were not included in the standard operating equipment of the study site during the study reference period. While the above mentioned assessment methodologies were considered as they are usually necessitated by the clinical setting during its standard operations for rapid, non-invasive longitudinal monitoring of patients, also for clinical evaluation purposes not necessarily related to research needs, a comprehensive evaluation of COPD+MPS patients trajectory warrants the integration of objective instrumental diagnostics; supplementing scales with spirometry, algometry, myorheological profiling, and biochemical analysis would facilitate a direct quantification of the physiochemical dysfunctions underpinning COPD and MPS. Although the selection of the assessment scales applied was unmodifiable and not prospective, it should be noted that the applied scales are all self-administered and independent of the administration skills of one or more researchers, thus reducing observer expectation bias and interviewer-related bias in the clinical assessment of patients [49]. Furthermore, since the assessments, as per the routine of the study center, were collected by internal medical staff, and not by the same balneotherapy practitioners performing the BPM-ITC protocol, the risk of bias in data collection was further limited. However, this does not mean that some data collection bias may not exist, such as due to specific, unknown psycho-emotional states present in patients at the time of completing the scales. This makes the use of more objective assessment tools even more advisable for any future studies on the topic.

Anyhow, the high level of patient adherence to BPM-ITC treatment and the lack of reports of related adverse events support its safety, tolerability, and replicability. Moreover, these positive characteristics have been found in cases with generally heterogeneous clinical and demographic characteristics, which could support its applicability in patients with different general features, comorbidities, and pathological severity, although this aspect should be investigated in larger populations to confirm this consideration.

Given the aforementioned limitations, the therapeutic mechanisms of BPM-ITC were purely elucidated based on established neurophysiological effects of balneotherapy-type interventions. To confirm the validity of these preliminary findings, subsequent studies must incorporate more comprehensive assessment tools. Future investigations conducted within a controlled research setting, with more accurate and specific evaluation tools, utilizing larger and more representative populations, and comparing the BPM-ITC approach to standard COPD care, are essential to confirm the clinical potential of the approach, as preliminarily observed in this study.

Although COPD rehabilitation guidelines are still generally heterogeneous and only moderately developed, as are those for MPS, the integration of multidisciplinary, multitherapeutic, and personalized approaches is believed to be the right clinical path to improve the management of these conditions [50,51]. Therefore, continuing research on rehabilitation approaches based on these characteristics, such as the BPM-ITC observed in our study, could help better define the most effective treatment pathways for the specific needs of each patient, especially when affected by overlapping pathological conditions of the viscero-somatic type.

## 5. Conclusions

BPM-ITC in patients with comorbid COPD and MPS seems to be associated with significant improvements in dyspnea severity, pain intensity, and bio-postural health. Furthermore, some factors such as gender, BMI, severity of comorbidities, and initial musculoskeletal symptoms appear to partially influence the responsiveness to BPM-ITC treatment, which, however, seems to be generally tolerable and free of adverse reactions manifested during or immediately after its application.

Given the high prevalence of both COPD and MPS, and their possible overlap, as well as their associated musculoskeletal and visceral sequelae, this non-invasive approach might offer a scalable complementary therapeutic option.

The high patient compliance and its applicability across diverse demographic profiles position BPM-ITC as a potentially valid tool for the clinical management of COPD+MPS patients.

However, further research is warranted to standardize application protocols within an evidence-based, multidisciplinary framework, consistent with the principles of translational medicine, in order to identify demographic factors that could influence the clinical outcome of patients undergoing the BPM-ITC protocol and better understand its potentially beneficial physiological effects on health.

## Figures and Tables

**Table 1:** Main demographic characteristics of the observed cases.

Pt.	Age (Years)	Sex	Height (cm)	Weight (kg)	BMI (kg/m^2^)	Comorbidities	NPRS	MRC	BPQ Score
1	78	Male	165	80	29.4	Cervical + Lumbar Spinal Stenosis	10	3	52
2	61	Male	166	80	29.0	Polyarthritis; Polyneuropathy	10	3	80
3	59	Female	165	69	25.3	Arthrosis	8	2	42
4	68	Female	162	62	23.6	Bilateral Coxarthritis; CVI; Breast Quadrectomy Outcomes	9	2	44
5	75	Female	161	67	25.8	Severe Polyarthritis	9.5	3	41
6	72	Female	160	70	27.3	Osteoporosis; Fibromyalgia; Cervicalgia; Bilateral Gonalgia; CVI	8.5	2	48
7	76	Female	157	61	24.7	Low Back Pain; Upper Back Pain; Spondyloarthritis; Metabolic Syndrome; CVI	6.5	2	30
8	76	Female	165	75	27.5	Low Back Pain; Mild Spondylosis; Lumbar Spinal Stenosis; Mild Hypertension	6	3	20
9	64	Male	168	85	30.1	Bilateral Sciatica; Obesity	6.5	2	27
10	63	Male	172	100	33.8	Stroke Outcomes; Mild Hypertension; Polyarthritis; Hypothyroidism; Obesity	10	2	40

Legend: Pt. = Patient; BMI = Body Mass Index; NPRS = Numeric Pain Rating Scale; MRC = Medical Research Council dyspnea scale; BPQ Score = Bio-Postural Questionnaire score; CVI = Chronic Venous Insufficiency.

**Table 2:** Values of the parameters observed for each patient at times T0 and T1, and statistical analysis of the variations.

Pt.	MRC		NPRS		BPQ Score	
	T0	T1	T0	T1	T0	T1
1	3	2	10	6	52	38
2	3	2	10	5	80	33
3	2	1	8	5	42	19
4	2	1	9	6	44	29
5	3	2	9.5	8	41	39
6	2	1	8.5	5	48	42
7	2	1	6.5	4.5	30	12
8	3	2	6	3	20	11
9	2	1	6.5	6	27	8
10	2	1	10	6	40	26
Mean	2.4	1.4	8.4	5.4	42.4	25.7
Median	2	1	8.7	5.5	41.5	27.5
IQR	1	1	3.5	1	18	26
*p*	<0.01		<0.01		<0.01	
r	−0.98		−0.87		−0.87	

Legend: Pt. = Patient; MRC = Medical Research Council dyspnea scale; NPRS = Numeric Pain Rating Scale; BPQ Score = Bio-Postural Questionnaire score; IQR = Interquartile Range; r = effect size. Note: Means and Medians rounded to one decimal place; *p* and r values rounded to two decimal places.

## Data Availability

The anonymized clinical data collected for this case series are fully reported within the present article in the Section 3.

## Abbreviations

The following abbreviations are used in this manuscript:

COPD	Chronic Obstructive Pulmonary Disease
MPS	Myofascial Pain Syndrome
MTrPs	Myofascial Trigger Points
MRC	Medical Research Council
NPRS	Numeric Pain Rating Scale
BPQ	Bio-Postural Questionnaire
Pt.	Patients
BMI	Body Mass Index
IQR	Interquartile Range
CVI	Chronic Venous Insufficiency
